# Implementing Core Entrustable Professional Activities in Emergency Medicine Clerkships: A Psychometric Study of Student Growth

**DOI:** 10.1002/aet2.70191

**Published:** 2026-06-23

**Authors:** Anne Marie Griebie, Esther Dasari Dale, Mohammed A. A. Abulela, Claudio Violato

**Affiliations:** ^1^ University of Minnesota Medical School Minneapolis Minnesota USA; ^2^ Department of Educational Psychology University of Minnesota Minneapolis Minnesota USA

## Abstract

**Background:**

The purpose of the present study was to conduct a psychometric analysis of student growth of Entrustable Professional Activities (EPAs) in an Emergency Medicine (EM) clerkship.

**Methods:**

A total of 481 third or fourth‐year medical students at the University of Minnesota Medical School participated. Students were enrolled in a required 4‐week EM clerkship during 3 academic years across 13 Core EPAs. Regression analyses were employed to examine if the growth curve of each of the EPAs follows a curvilinear structure as hypothesized in learning theory. Reliability or dependability of the EPA assessments was assessed using two‐facet generalizability analysis defined as Ep2 coefficients indicating the reliability of the scores.

**Results:**

There were 7707 EPA‐based assessments (mean = 16 [SD = 5.0] assessments per student), provided by 793 assessors; 494 (62.3%) residents and 299 (37.7%) faculty members. The growth curves for most EPAs ratings followed the predicted negative exponential curve (EPAs 1, 2, 3, 5, 6, 8, 10, 12) but not for others (4, 7, 9, 11, 13). The slope of the growth curves showed variation by EPA. Adequate reliability of ratings was achieved with six raters assessing on six assessment occasions (Ep2 coefficient ≥ 0.70).

**Conclusions:**

Analyses of more than 7700 assessments of students in an emergency medicine rotation are consistent with long‐standing negative exponential learning theory. Adequate reliability was achieved with an acceptable Ep2 coefficient with six assessors. Results indicate the EPA framework is a practical approach to assessing real‐world competency within the emergency medicine clerkship. Moreover, student growth on these EPAs provides important information for clerkship design, instruction, and assessment.

## 1 Introduction

### 1.1 Background

Educational leaders such as the Association of American Medical Colleges (AAMC) have noted a need for a consistent assessment framework across both undergraduate medical education (UME) and graduate medical education (GME) to standardize student achievement both longitudinally and cross‐institutionally [1]. To facilitate an outcomes‐based approach (Competency‐Based Medical Education or CBME) [2, 3], the AAMC convened an expert panel to develop a model of activities that residents should be able to perform on day one of their residency training regardless of specialty. These have become known as the 13 Core Entrustable Professional Activities for residency (Core EPAs) [4, 5]. These behavioral tasks are “units of professional practice, defined as tasks or responsibilities to be entrusted to the unsupervised execution by a trainee once he or she has attained sufficient specific competence” [2]. They provide the contextual performance for which student competency of clinical skills for entering residency can be assessed.

Educational frameworks such as the Core EPA system can enhance the consistency and reliability of ratings with various assessment tools. Medical education is broad yet retains an overarching goal of training competent and trustworthy physicians, thus increasing consistency across institutions which leads to better evaluation and attainment of this shared goal. While several medical education assessment frameworks have been proposed, such as the National Undergraduate Framework in the Netherlands [6] and the Canadian Medical Education Directions for Specialists (CanMEDS) [7], a most promising educational and assessment framework is the Core EPA system [8]. Practice accomplishing the associated tasks begins at different stages of undergraduate medical education across different curricula. Nonetheless, the overall goal across institutions utilizing the Core EPAs is to provide feedback and assessment to students in the clerkship phase of their medical education.

Analyzing existing Core EPA data regarding growth curves allows for a better understanding of the time it takes students to reach a given competency level, such as entrustment. Such data can also provide construct validity evidence of the EPA system as it demonstrates performance consistent with a theoretically predictable pattern [9, 10]. The growth curve currently found to be most consistent with the predicted developmental growth pattern of EPA attainment demonstrates a learning pattern known as negative exponential learning theory. This theory posited by Thurstone [11] suggests learning gains are steep initially and eventually plateau. Several studies indicate student competence across the Core EPAs follows this predicted negative exponential growth [9, 10]. Other studies similarly suggest EPA attainment follows a nonlinear trajectory with increased ratings, providing construct validity evidence in favor of utilizing an EPA assessment framework and differences in student growth curves [12].

Dambro et al. [13] highlighted how residency program directors have begun placing a higher weight on the Medical Student Performance Evaluations (MSPE) portion of residency applications but continue to have concerns about the validity, objectivity, and standardization of the EPAs. Residency program across various specialties see EPAs use as favorable overall. Various challenges and differences in perceptions remain, however [14, 15, 16]. A potential future transition toward more standardization of EPA assessment across UME institutions may improve the perception and usefulness of CBME programs and outcomes [14].

Despite wide interest in valid student assessment, further empirical research is required to evaluate the validity of the Core EPA system in UME. Several studies provide evidence in support of the Core EPAs by demonstrating that medical student competence during core‐clerkship rotations follows an expected nonlinear developmental assessment model consistent with negative exponential learning theory [9, 10, 11]. The AAMC suggests several EPAs (3, 4, 8) may be outside the scope of traditional clerkship duties and better suited for evaluation during rotations taken later in more senior UME training [10, 17, 18, 19]. Evidence also suggests students are challenged to reach high levels of attainment on several EPAs (8, 11) at the beginning stages of their clerkship education. Moreover, sub‐internship level rotations lead to a large perceived increase in EPA competency among undergraduate medical students [20]. Currently, there is little empirical evidence exploring the validity of the 13 Core EPAs in assessing later stages of clinical immersion, including in emergency medicine clerkships.

### 1.2 Importance

Evaluating the validity of the 13 Core EPAs for use in UME emergency medicine clerkship is especially important as other specialty‐specific assessment frameworks have been proposed, including the National Clinical Assessment Tool for Medical Students in Emergency Medicine (NCAT‐EM) [21, 22] which shows significant inter‐institutional variability in scoring [22] and minimal reliability across student cohorts [21]. Given the Core EPAs are essential tasks a medical student should be trusted to perform with indirect supervision on entrance to residency, they are most useful for formative assessment if used consistently across both rotations and institutions. Thus, it is important to close this research gap and examine the growth and entrustability of the 13 Core EPAs for students within the emergency medicine rotation specifically.

### 1.3 Goals of This Investigation

In the present study we seek to close the research gap on the validity of the 13 Core EPAs for use in UME emergency medicine clerkship by analyzing the growth of EPA scores, modeling the learning curves of each of the 13 EPAs, and calculating the reliability or dependability of EPA ratings.

## 2 Method

### 2.1 Selection of Participants

A total of 481 (271 women—56%; 210 men—44%) third or fourth‐year medical students at the University of Minnesota Medical School, with mean age = 24.3 (SD = 2.6) at matriculation, participated. Students were enrolled in a required 4‐week Emergency Medicine (EM) clerkship during 3 academic years 2021–2024. Students typically complete the EM clerkship late in their clinical rotation schedule, with most students completing the clerkship during their fourth‐year.

### 2.2 Data Collection and Procedures

A total of 793 assessors provided at least one EPA rating in the data analyzed for this study: 494 (62.3%) residents and 299 (37.7%) faculty members. Ratings from all 13 Core EPAs were included in the current analyses. The mean number of ratings per assessor was 9.7 (SD = 12.93; range: 1–129).

Students were assessed during their EM required clerkship on the 13 Core EPAs described in Table 1. They were required to obtain EPA assessments provided by clinical faculty, residents supervising the students, or Assessment and Coaching Experts (ACEs). The ACEs are a group of 15 faculty members trained in competency‐based assessment, in general, and in the Core EPA assessment specifically. The ACEs serve as faculty developers of the EPAs and educate both faculty and residents on standardized EPA assessment through both direct observation and longitudinal monitoring to reduce subjectivity in evaluation. Additionally, all raters had access to educational materials regarding EPA assessment.

**TABLE 1:** Descriptive statistics for entrustable professional activities (EPAs), regression goodness‐of‐fit (*R* ^2^), and slope of the growth curves.

Entrustable professional activities	Total # of ratings	Mean # ratings/student	*R* ^2^ ^a^	*β*_0_ (slope^b^)
EPA 1: Gather a history and perform a physical examination	997	2.07	0.87	0.072*
EPA 2: Prioritize a differential diagnosis following a clinical encounter	1007	2.09	0.90	0.084*
EPA 3: Recommend and interpret common diagnostic and screening tests	395	0.82	0.65	0.117**
EPA 4: Enter and discuss orders and prescriptions	178	0.37	0.34	0.043
EPA 5: Document a clinical encounter in the patient record	244	0.51	0.36	0.153**
EPA 6: Provide an oral presentation of a clinical encounter	586	1.22	0.95	0.097*
EPA 7: Form clinical questions and retrieve evidence to advance patient care	69	0.14	0.11	−0.049
EPA 8: Give or receive a patient handover to transition care responsibility	542	1.12	0.97	0.177**
EPA 9: Collaborate as a member of an interprofessional team	175	0.36	0.06	0.061
EPA 10: Recognize a patient requiring urgent or emergent care and initiate evaluation	2346	4.87	0.81	0.048*
EPA 11: Obtain informed consent for tests and/or procedures	296	0.61	0.04	0.019
EPA 12: Perform general procedures of a physician	620	1.29	0.71	0.109*
EPA 13: Identify system failures and contribute to a culture of safety and improvement	252	0.52	0.57	0.101

^a^ *R* ^2^ is a fit index for the regression line to the raw data—0 to 1; 0 = no fit, 1 = perfect fit.

^b^ The slope (***β***_0_) of the negative exponential (e) EPA growth curves of the form, *Y* = ***β***_0_ *X* ^e^ + *c*. The larger the value ***β***_0_, the steeper the learning curve (Figure 1).

* < 0.01.

** < 0.001.

To obtain a rating, a student who has completed an interaction with a patient will ask their supervisor to provide a rating on one (or more) of the 13 EPAs. The student accesses a link to the EPA rating form on their mobile phone and passes their phone to the supervisor of their choosing to provide the rating (or has the supervisor tell them which rating to input into the form). Each assessment requires the supervisor to rate the student on a scale of 1–5: (1) Observation only: “I [resident, faculty member] did it. The student observed,” (2) Direct Supervision: “We did it together,” (3) Direct Supervision: “I supervised and helped the student from time to time,” (4) Indirect Supervision: “The student did it. I double‐checked ALL elements,” (5) Indirect Supervision: “The student did it. I double‐checked KEY elements.” [10, 23] Scores of 4 or greater meet the desired threshold for entrustment. Additionally, assessors provide narrative feedback on one area of strength and one area that could be improved to help the student improve their clinical skills. Each student was required to receive at least 12 EPA ratings over the course of their 4‐week clerkship.

### 2.3 Analyses

The units of analysis are assessments completed on each of the 13 Core EPAs. Strictly speaking, the entrustment rating 5‐point scale is ordinal, similar to the widely used Likert scale. An analytical approach employed for these scales is based on the assumption they are interval or “quasi‐interval.” Numerous Monte Carlo studies support using these parametric approaches in the field of ordinal analysis [24]. The resulting estimate of the population parameter is almost always the best in the sense of being closest to the true value [25]. Student ratings were depicted as curves describing their progress on the 5‐point entrustment scale over time; regression models were employed to fit the theoretical curves to the actual data.

### 2.4 Regression Analyses

Since the primary purpose of these analyses was to examine the growth of the students throughout their EM rotation, we conducted regression analyses to assess the relationship between the time variable (week during the rotation; i.e., Week 1–4) and students' average ratings on the entrustment scale. We wished to examine if the growth curve of each of the EPAs would demonstrate a curvilinear structure (e.g., negative exponential), as hypothesized by Thurstone [11]. Thus, we examined curvilinear relationships at the group level, employing random effects analysis.

### 2.5 Reliability Analysis: Generalizability

We employed a two‐facet (occasion × assessor) nested design [Student * (F1: F2)] to derive G‐coefficients for the reliability or dependability of the EPA assessments. A sample (*n* = 68) that had all been assessed on 6 occasions on EPA 10 was selected for the G‐theory analysis [26].

### 2.6 Ethical Approval

The University of Minnesota‐Twin Cities institutional review board determined this study to be exempt from review.

## 3 Results

### 3.1 Characteristics of Study Subjects

A total of 7707 EPA‐based assessments were done for the 481 students (mean number of assessments per student = 16; SD = 5.0) across three academic years (2021–2022; 2022–2023; 2023–2024) of the required EM clerkship. Table 1 summarizes descriptive statistics for each EPA, and the results of the regression analyses showing the slope of the growth curves and a goodness‐of‐fit (*R* ^2^).

### 3.2 Main Results

The largest number of assessments was for EPA 10 (Recognize a patient requiring urgent or emergent care and initiate evaluation: 2346; mean = 4.87 per student), followed by EPA 1 (Gather a history and perform a physical examination: 997; mean = 2.07 per student) and EPA 2 (Prioritize a differential diagnosis following a clinical encounter: 1007; mean = 2.08 per student). Several EPAs had low number of ratings per student (Table 1) such as EPA 7 (Form clinical questions and retrieve evidence to advance patient care: 69; mean = 0.14), EPA 11 (Obtain informed consent for tests and/or procedures: 296; mean = 0.61), and EPA 13 (Identify system failures and contribute to a culture of safety and improvement: 252; mean = 0.52). These three EPAs with the low number of ratings per student can be grouped into EPA duties more typically aligned with sub‐internship/acting internship curriculum (EPA 7) and EPAs more typically aligned with duties reserved for interns/residents (EPA 11, 13) [8, 27].

The penultimate and final columns in Table 1 contain the goodness‐of‐fit index (R [2]) and the slope of the growth curve of each EPA, respectively.

### 3.3 Growth (Learning Curves)

**FIGURE 1:**
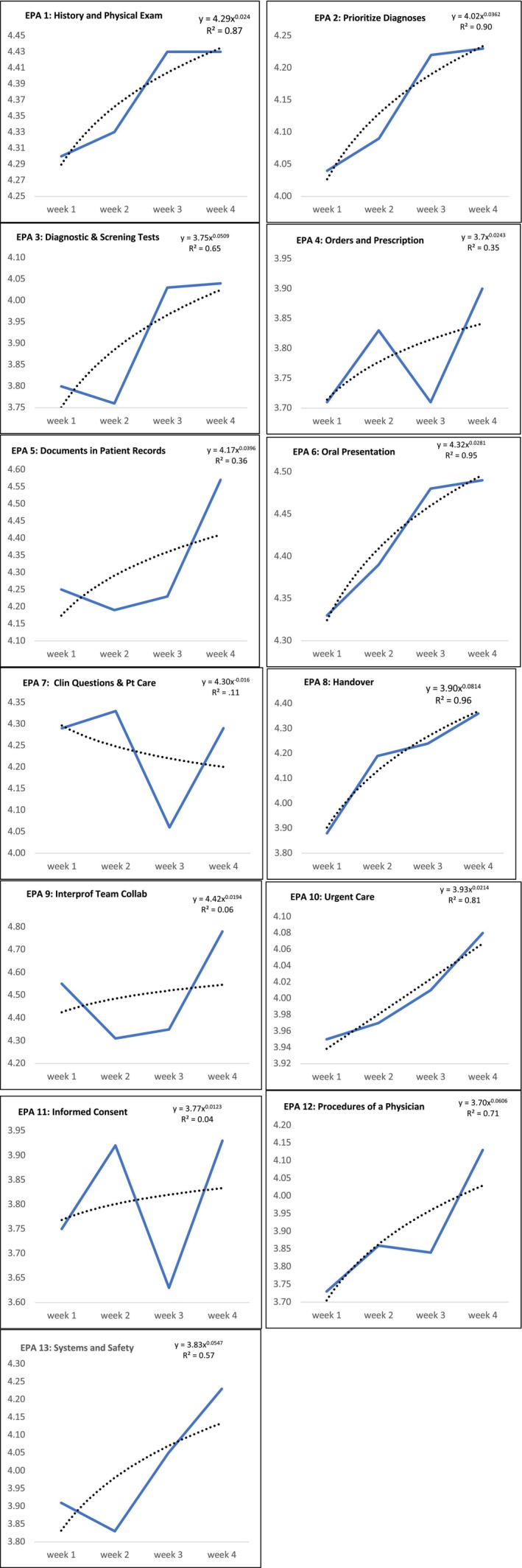
Entrustable professional activity (EPA) growth curves for third or fourth‐year medical students at the University of Minnesota Medical School from three academic years. An EPA score of 4 is considered entrustable. Solid line indicates raw data; dotted line indicates fitted curve. Mean EPA score is represented by the *y*‐axis. EM clerkship week is represented by the *x*‐axis.

Visual displays of EPA growth are presented as learning curves in Figure 1 showing the raw data growth trajectories for all students combined during the 4‐week period of the EM rotation. The theoretically fitted curves are also superimposed on the raw data and demonstrate curvilinear relationships between time and EPA ratings (*p* < 0.05 for EPAs 1,2,3,5,6,8,10,12 but nonsignificant for 4,7,9,11,13—see Figure 1).

Close inspection of these figures demonstrates learning curves as students approach and exceed a rating of 4 on the supervision scale (the desired score for entrustment by the time of graduation), defined as: “is allowed to practice EPA only under reactive/on‐demand supervision available and all findings double checked.” In many instances, the students even approached a rating of 5, which is defined as: “is allowed to practice EPA only under reactive/on‐demand supervision available and key findings double checked.”

### 3.4 Reliability Analysis (G‐Theory)

**FIGURE 2:**
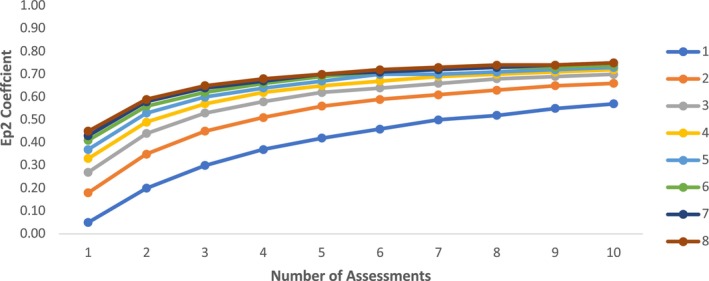
Generalizability Coefficient (Ep2) by Number of Assessors and Number of Assessments (EPA10—Urgent Care). The different colored lines represent different numbers of assessors.

We adapted SPSS algorithms for these analyses based on the work of Mushquash and O'Connor [27], using a two‐facet (occasion × assessor) nested design [Student * (F1: F2)] to derive G‐coefficients reliability or dependability calculations with a sample (*n* = 68) that had all been assessed on at least 6 occasions on EPA 10. Adequate reliability or dependability (Ep2 ≥ 0.70) required 6 assessments conducted by 6 raters (see Figure 2).

## 4 Discussion

The present study employed rating data on EPAs gathered from undergraduate medical students in an emergency medicine 4‐week rotation to (1) analyze the growth of EPA scores, (2) model the learning curves of each of the 13 EPAs, and (3) calculate the reliability or dependability of EPA ratings.

Based on previous work [9, 10], we posited students' learning curves would follow Thurstone's theory of negative exponential growth, thus providing evidence of construct validity [9, 10, 11]. Both the raw data plotted over time and the fitted curves followed a negative exponential function for 8 of the EPAs (1–3, 5, 6, 8, 10, 12) but not for 5 others (4, 7, 9, 11, 13). The model fit indices (Table 1) are very high for the 8 negative exponential function EPAs (*R* ^2^ range: 0.71–0.97). The poorer model fit for the other 5 EPAs is likely due to the small number of ratings (69 for EPA 7 to 296 for EPA 11) over the 3 academic years resulting in a mean number of ratings per student of less than 1. These low numbers of ratings result in under‐powered analyses leading to poor model fit (*R* ^2^ range: 0.06–0.57). This interpretation is supported by previous findings where growth for all 13 EPAs followed a negative exponential function with larger numbers of ratings and, therefore, adequately powered analyses [10]. Accordingly, the results of the present study provide evidence supporting the construct validity of EPAs as an assessment system of professional growth.

The present results confirmed growth across EPAs is variable. The rate of growth as indicated by the slope of the curves and the model fit is in concordance with theoretical expectations due to student, EPA, and curriculum factors [28]. Previous work, for example, suggested EPA 10 (Recognize a patient requiring urgent or emergent care and initiate evaluation) is assessed predominantly in the emergency room, and EPA 8 (Give or receive patient handover to transition care responsibility) is primarily assessed in more senior UME rotations including the Acting Internship in Critical Care rotation in 4th year [10]. The present study confirms these results; EPA 10 had the highest number of EPA assessments obtained across all students, and both EPA 8 and 10 showed high rates of learning and growth. Overall, there were rapid growth rates for EPAs 1,2,6,10,12, moderate growth for 4,5,7,9,11 and minimal growth for 3, 13 (Table 1 and Figure 1).

EPA factors influencing growth include complexity and ease of observation. Inadequate opportunities for task‐specific practice may also influence the growth rate on certain EPAs within the curriculum. Past research suggests students have little opportunity to obtain informed consent for tests and procedures (EPA 11), and did not seek opportunities to identify systems failure and contribute to a culture of safety and improvement (EPA 13) [8]. The present results confirm this and also suggest lower rates of assessments obtained for these two EPAs. Ongoing effort should be dedicated to ensuring there is sufficient time and curriculum/educational experiences in areas where learning, assessment, and growth are more difficult to assess or attain.

Residency program directors and new residents have reported the least confidence in the new resident ability for EPA 4 (Enter and discuss orders and prescriptions) and EPA 13 (Identify system failures and contribute to a culture of safety and improvement) [14, 29]. These reports align with our results showing little growth for EPA 4 and 13 for our sample. Both of these EPAs are regarded as necessary for new residents, thus suggesting the need for some curriculum change to allow for improved student learning and assessment of these activities [14]. Implementing such change will be challenging; perhaps such curriculum changes can be achieved through simulated scenarios or an additional course to learn about quality improvement within the healthcare system. Additionally, it may be worth considering whether some of the behaviors described by the Core EPAs are appropriate to expect medical students to obtain prior to entering residency. Continued evaluation of the usefulness of these behaviors and need for revision is warranted.

Reliability analysis for a particularly important EPA for emergency medicine (EPA 10: Recognizing urgent care) was conducted using G‐theory. Rigorous reliability analysis is particularly important as the accuracy of EPA assessments continues to be an issue. The results indicate adequate dependability of the scores is achieved with 6 raters conducting 6 assessments. In similar previous analyses with EPA 1 (History and Physical Exam), we found adequate reliability could be achieved with 4 raters conducting 4 assessments [10]. The reliability of the EPA scores may vary somewhat across EPAs, but dependability of scores can be achieved with 4–6 raters on 4–6 assessment occasions. Further research on dependability is required.

### 4.1 Limitations and Strengths

There are some limitations of the current study. We focused on the EM clerkship, which provides clinical learning opportunities with increased time pressure and bedside teaching rather than formal rounding. While all of these features make the emergency department a good setting for assessing the validity of the EPA system, the generalizability of the current findings for other core clerkship rotations may be limited. Also, this was a single institution study (UMMS) and may be limited in its generalizability to other medical schools. Additionally, in this study, learning curves were not analyzed at the individual student level, but instead at an aggregate level. It is likely individual student factors have some influence on individual EPA attainment. Finally, it is possible different raters (clinical faculty, residents, ACEs) rated students differently based on experience and familiarity with EPAs; further exploration into rater differences could prove useful.

Nonetheless, the present study does have some strengths: (1) a large sample of students (481) assessed during clinical immersion, (2) a large number of assessments overall (7707), (3) a multiyear study (over 3 academic years), and (4) sophisticated, comprehensive psychometric analyses for validity and reliability evidence.

## 5 Conclusions

The results of Core EPA assessments for Emergency Medicine are in accordance with negative exponential learning theory and provide useful data to measure growth of medical students' clinical skills. The Core EPA framework can accurately capture students' expected growth within a 4‐week EM rotation. The results of our analyses of more than 7700 assessments over 3 years in the undergraduate clinical immersion of EM are consistent with long‐standing learning theory. Our overall results indicate the Core EPA framework is a practical approach to assessing real‐world competency within the EM clerkship. Moreover, student growth on these EPAs continues to provide important information for course design, instruction and assessment.

## Author Contributions

**Claudio Violato:** conceptualization, writing – review and editing, supervision, formal analysis, project administration, data curation, investigation. **Esther Dasari Dale:** conceptualization, data curation, supervision, writing – review and editing, project administration, investigation, formal analysis. **Mohammed A. A. Abulela:** formal analysis, writing – review and editing, data curation, investigation.

## Funding

The authors have nothing to report.

## Disclosure

The authors have nothing to report.

## Ethics Statement

This study was approved as exempt by the University of Minnesota Medical School Institutional Review Board.

## Conflicts of Interest

The authors declare no conflicts of interest.

## Data Availability

The data that support the findings of this study are available on request from the corresponding author. The data are not publicly available due to privacy or ethical restrictions.
